# Seasonal Patterns in Human A (H5N1) Virus Infection: Analysis of Global Cases

**DOI:** 10.1371/journal.pone.0106171

**Published:** 2014-09-12

**Authors:** Maya B. Mathur, Rita B. Patel, Michael Gould, Timothy M. Uyeki, Jay Bhattacharya, Yang Xiao, Yoshi Gillaspie, Charlotte Chae, Nayer Khazeni

**Affiliations:** 1 Quantitative Sciences Unit, Stanford University Department of Medicine, Stanford, California, United States of America; 2 Division of Pulmonary and Critical Care Medicine, Stanford University Medical Center, Stanford, California, United States of America; 3 Kaiser Permanente Southern California, Pasadena, California, United States of America; 4 Influenza Division, National Center for Immunization and Respiratory Diseases, Centers for Disease Control and Prevention, Atlanta, Georgia, United States of America; 5 Center for Health Policy and Center for Primary Care and Outcomes Research, Stanford University, Stanford, California, United States of America; 6 Department of Languages, Literatures, and Cultures, University of South Carolina, Columbia, South Carolina, United States of America; Arizona State University, United States of America

## Abstract

**Background:**

Human cases of highly pathogenic avian influenza (HPAI) A (H5N1) have high mortality. Despite abundant data on seasonal patterns in influenza epidemics, it is unknown whether similar patterns exist for human HPAI H5N1 cases worldwide. Such knowledge could help decrease avian-to-human transmission through increased prevention and control activities during peak periods.

**Methods:**

We performed a systematic search of published human HPAI H5N1 cases to date, collecting month, year, country, season, hemisphere, and climate data. We used negative binomial regression to predict changes in case incidence as a function of season. To investigate hemisphere as a potential moderator, we used AIC and the likelihood-ratio test to compare the season-only model to nested models including a main effect or interaction with hemisphere. Finally, we visually assessed replication of seasonal patterns across climate groups based on the Köppen-Geiger climate classification.

**Findings:**

We identified 617 human cases (611 with complete seasonal data) occurring in 15 countries in Southeast Asia, Africa, and the Middle East. Case occurrence was much higher in winter (n = 285, p = 0.03) than summer (n = 64), and the winter peak occurred across diverse climate groups. There was no significant interaction between hemisphere and season.

**Interpretation:**

Across diverse climates, HPAI H5N1 virus infection in humans increases significantly in winter. This is consistent with increased poultry outbreaks and HPAI H5N1 virus transmission during cold and dry conditions. Prioritizing prevention and control activities among poultry and focusing public health messaging to reduce poultry exposures during winter months may help to reduce zoonotic transmission of HPAI H5N1 virus in resource-limited settings.

## Introduction

With a case-fatality proportion of approximately 60% [1], highly pathogenic avian influenza (HPAI) A (H5N1) virus is a serious public health threat in a number of countries. Human cases of HPAI H5N1 virus infection have been reported from 15 countries in Asia, Africa, and the Middle East. Many of these countries have limited healthcare resources [2], and HPAI H5N1 is causing substantial health, social, and economic burdens in poultry and agricultural sectors [3]–[6].

Despite abundant research on seasonal fluctuations in annual epidemic influenza activity worldwide, it is unknown whether similar patterns exist in human cases of HPAI H5N1. Isolates from aquatic and terrestrial poultry in mainland China were most frequently HPAI H5N1-positive during winter [7], [8], and drops in temperature tend to immediately precede avian outbreaks [9]. Such seasonal patterns among avian vectors suggest similar patterns might exist in human cases. While there is some evidence of seasonal patterns of human HPAI H5N1 virus infection in Egypt [10] and Indonesia [11], we are not aware of a comprehensive, global analysis using all known human cases to date.

We set out to determine whether the epidemiology of human cases of HPAI H5N1 follows temporal patterns similar to seasonal influenza, aiming to provide this data to increase prevention and control measures among poultry and public health prevention messages about vigilance near birds during seasons with higher risk of human infections. We investigated seasonal patterns in the occurrence of human cases of HPAI H5N1 using a comprehensive data set comprising all confirmed, symptomatic cases published in the literature since the initial 1997 outbreak in Hong Kong. We assessed hemisphere as a potential moderator of seasonal patterns, since seasonal incidence patterns due to weather changes could be dependent on hemisphere. Due to the tilt of the Earth's rotational axis, seasonal weather patterns are reversed between the Northern and Southern hemispheres, with Equatorial regions showing less seasonal variation [12]. Finally, we assessed the graphical similarity of seasonal patterns across six diverse climate types.

## Methods

### Systematic Literature Search

To compile a dataset of laboratory-confirmed and possible human cases of HPAI H5N1 worldwide, we systematically searched four databases: PubMed, Scopus, Google Scholar, and the World Health Organization Global Alert and Response compilation (WHO GAR) [2]. We created an initial dataset using cases published in the WHO GAR website, which includes cases reported since November 2003. We assumed laboratory confirmation satisfying the WHO reporting standard for all such cases, although specific clinical laboratory data were not provided.

We then reviewed articles published in PubMed, Scopus, and Google Scholar using keywords “H5N1” and “human” or “humans.” We included articles in any language published between January 1, 1997 (the year of the initial Hong Kong outbreak) and April 19, 2013. We included articles describing confirmed or possible human cases of HPAI H5N1 virus infection. We defined confirmed cases as those meeting WHO reporting criteria: isolation of HPAI H5N1 virus, a positive result by reverse transcription polymerase chain reaction (RT-PCR) testing of clinical specimens using H5-specific primers and probes, an elevated H5-specific antibody titer of ≥1∶80 (or equivalent using the WHO protocol), or at least a fourfold rise in H5N1 virus neutralization antibody titer in paired sera [13]. We defined possible cases as those lacking laboratory confirmation but exhibiting symptoms and having known exposure to a confirmed human case. Due to limited availability of data on other modes of transmission (such as via consumption of raw poultry meat or exposure to feces-contaminated environments), this definition is more restrictive than the WHO definitions of suspected and probable cases. Two independent reviewers (RP, MM) assessed each article for these inclusion criteria; a third reviewer (NK) resolved disagreements.

For articles in Japanese, Russian, French, and Spanish, we verified inclusion with native speakers. A professional translator (YX) assessed inclusion criteria for the large number of Chinese articles. For all other languages, we converted articles to text format using PDF OCR X software (version 1.9.32, Burnaby, British Columbia), then used an online translation program to translate the article to English and verify inclusion [14].

For articles meeting inclusion criteria, we added each reported case to our dataset, abstracting a predefined set of variables (Abstraction Form S1). To avoid duplicate entries, we systematically compared demographic data on each case to all others in our dataset.

### Variables

For each case, we extracted data on location (country and city) and date (month and year) of illness onset. We created a season variable based on month of case occurrence (defining summer as June–August, fall as September–November, winter as December–February, and spring as March–May). Based on country, we also defined a hemisphere variable (Northern or Equatorial; no Southern Hemisphere cases have been reported to date).

We defined a climate variable characterizing the predominant climate of the patient's city or region. We used the Köppen-Geiger climate classification map, which assigns one of 31 classifications incorporating three dimensions of climate: main climate (equatorial, arid, warm temperate, snow, polar), precipitation, and temperature [15]. The map is based on extensive data from the second half of the 20^th^ century, collected by the Global Precipitation Climatology Centre at the German Weather Service and the Climatic Research Unit of the University of East Anglia. For a small number of cases missing city or region data, but occurring in a country with a clearly predominant climate (n = 7), we imputed the country's predominant climate.

### Statistical Analysis

We performed all statistical analyses in R (Version 3.0.0, Vienna, Austria) and defined statistical significance by an alpha level of 0.05. Our primary analytic goal was to assess seasonal patterns in occurrence (frequency) of human cases of HPAI H5N1.

We first computed the proportion of cases occurring in each season. To assess significance of seasonal incidence patterns and to consider hemisphere as a moderator, we modeled case incidence using negative binomial regression. While the more common Poisson regression model is a canonical choice for modeling rare event occurrence, it relies on the assumption that the mean and variance of event frequency per unit time are equal. A common violation of this distributional assumption occurs when event frequency is over-dispersed: that is, the variance greatly exceeds the mean [16].

Negative binomial regression is a generalization of the Poisson model that allows a variance term to be fit to the data independent of the mean term, thus better modeling over-dispersion [16]–[18]. We note, however, that because Model 1 includes only orthogonal categorical factors, the maximum likelihood estimation process of any generalized linear model (GLM) in this case reduces mathematically to fitting separate means to each disjoint subset (defined by seasons). Thus, here, the choice of GLM is consequential only to the fitted standard errors and resulting p-values, not to the coefficients or predicted values.

We fit three nested models:
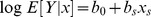
(1)

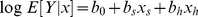
(2)

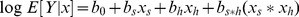
(3)where *Y* is case frequency, *b*_0_ is the intercept, *x_s_* is season (summer, fall, winter, spring) and *x_h_* is hemisphere (Northern, Equatorial). Thus, Model (1) allows us to assess the existence of seasonal patterns. We computed the incidence rate ratio (IRR) for fall, winter, and spring relative to summer by exponentiating their regression coefficients. Analogously to relative risk, the IRR represents the predicted multiplicative increase in event frequency for a covariate versus the reference level. That is, an IRR of 2 represents a twofold increase in event rate. As a secondary, post-hoc analysis, we used Akaike's Information Criterion (AIC) [19], a measure of fit-complexity tradeoff, and the likelihood-ratio test to compare Model (1) to Models (2) and (3), allowing us to assess the presence of a main effect of hemisphere and interaction of season with hemisphere. AIC is an entropy-based measure that decreases as the model becomes simpler (fewer parameters) and better-fitting (higher log-likelihood): thus, models with lower AIC are preferable.

Because cases have occurred in only 15 countries, with 55% of cases occurring in Egypt and Indonesia combined, inherent power limitations made quantitative assessment of climate type effects unfeasible. We instead present graphical representations as a preliminary analysis tool. We also manually synthesized the data we had compiled on HPAI H5N1 case occurrence with monthly weather data to visually assess the relationship between disease occurrence and local temperature, humidity, and season.

## Results

### Sample Characteristics

**Figure 1:**
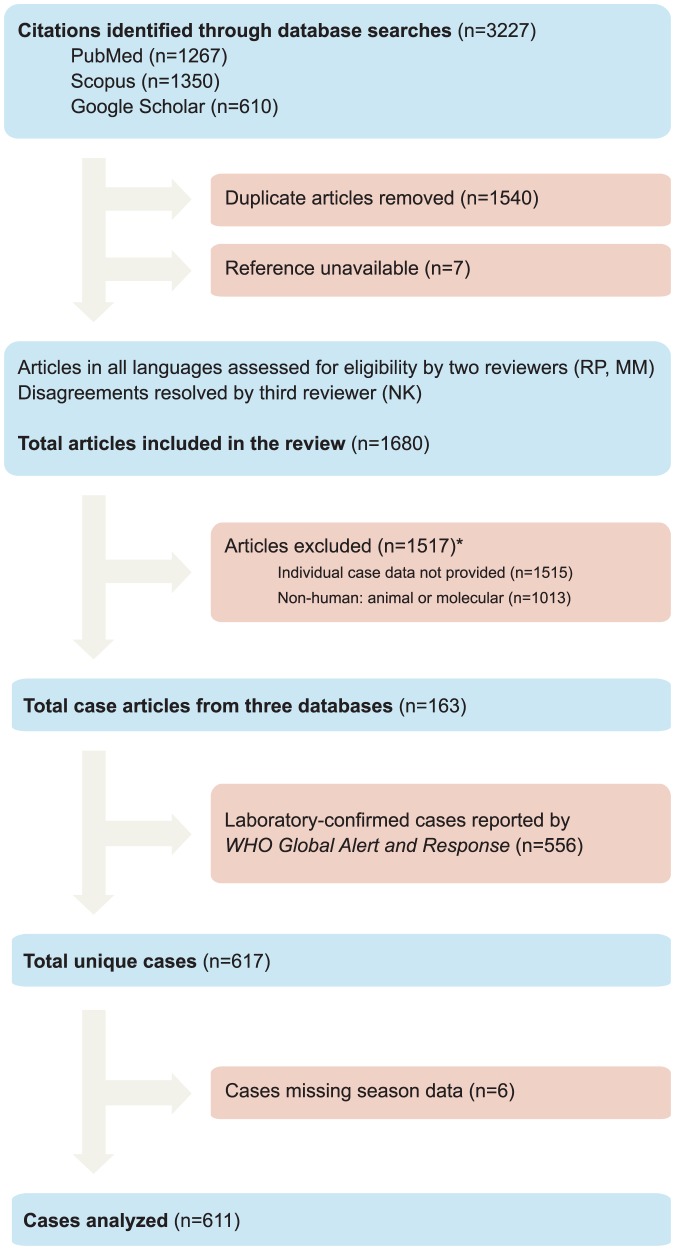
Literature search strategy. *: Total number of excluded articles is less than the sum of articles excluded by each criterion because most articles failed multiple criteria.

Our literature search identified 617 human cases of HPAI H5N1; we excluded from analysis six cases with missing data on season, yielding a final sample size of 611 (Figure 1). Cases occurred in 12 Northern Hemisphere countries and three equatorial countries (Table 1). Of note, all but two of the cases in equatorial countries were in Indonesia; the remaining two were in Nigeria and Djibouti.

**Table 1:** Seasonal distribution of highly pathogenic avian influenza (HPAI) H5N1 case occurrence by country, 1997–2013.

	Summer	Fall	Winter	Spring	*Total*
**Southeast Asia**					
Indonesia^e^	29 (17%)	37 (22%)	56 (33%)	49 (29%)	171
Vietnam	11 (12%)	3 (3%)	66 (73%)	11 (12%)	91
Cambodia	2 (6%)	1 (3%)	14 (39%)	19 (53%)	36
Thailand	4 (15%)	10 (37%)	12 (44%)	1 (4%)	27
Bangladesh	0 (0%)	0 (0%)	4 (67%)	2 (33%)	6
Laos	0 (0%)	0 (0%)	2 (100%)	0 (0%)	2
Myanmar	0 (0%)	1 (100%)	0 (0%)	0 (0%)	1
**China**					
China	2 (4%)	8 (16%)	33 (66%)	7 (14%)	50
Hong Kong	0 (0%)	9 (31%)	19 (66%)	1 (3%)	29
**Middle East**					
Egypt	16 (9%)	11 (7%)	64 (38%)	78 (46%)	169
Turkey	0 (0%)	0 (0%)	10 (100%)	0 (0%)	10
Azerbaijan	0 (0%)	0 (0%)	2 (22%)	7 (78%)	9
Pakistan	0 (0%)	5 (100%)	0 (0%)	0 (0%)	5
Iraq	0 (0%)	0 (0%)	2 (67%)	1 (33%)	3
Djibouti^e^	0 (0%)	0 (0%)	0 (0%)	1 (100%)	1
**Sub-Saharan Africa**					
Nigeria^e^	0 (0%)	0 (0%)	1 (100%)	0 (0%)	1
*Total*	*64 (10%)*	*85 (14%)*	*285 (46%)*	*177 (29%)*	*611*

Row percentages are reported.

e : country was classified as Equatorial (all others classified as Northern hemisphere).

**Figure 2:**
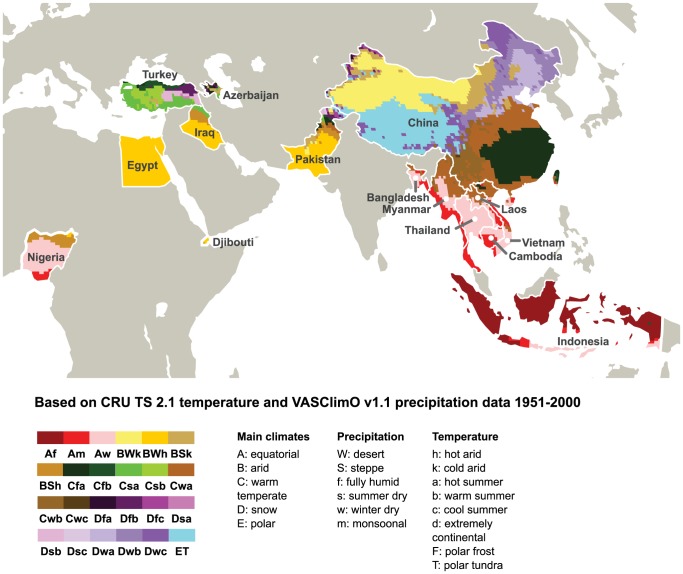
Köppen-Geiger climate classifications of highly pathogenic avian influenza H5N1 virus-affected countries. All countries with human HPAI H5N1 cases are color-coded based on climate classifications. Countries in gray have not yet reported human cases. We present an abbreviated color legend for clarity, showing only climate types occurring in HPAI H5N1-affected countries.

We found n = 591 cases with data for both climate and season. Cases occurred in regions representing 11 climate types, with Arid/desert/hot arid (n = 170) and Equatorial/fully humid (n = 144) being the most frequent climate types. Fewer than 90 cases occurred in each of the other nine climate groups (Table 2, Figure 2).

**Table 2:** Seasonal distribution of highly pathogenic avian influenza H5N1 case occurrence by climate type.

	Summer	Fall	Winter	Spring	*Total (n = 591)*
**Equatorial**					
Af (Equatorial/fully humid)	25 (17%)	30 (21%)	49 (34%)	40 (28%)	144
Aw (Equatorial/winter dry)	11 (13%)	13 (16%)	49 (60%)	10 (12%)	83
Am (Equatorial/monsoonal)	2 (4%)	5 (9%)	21 (39%)	26 (48%)	54
**Arid**					
BWh (Arid/desert/hot arid)	16 (9%)	11 (6%)	64 (38%)	79 (46%)	170
BSk (Arid/steppe/cold arid)	0 (0%)	0 (0%)	15 (68%)	7 (32%)	22
BWk (Arid/desert/cold arid)	1 (50%)	0 (0%)	1 (50%)	0 (0%)	2
**Warm-temperate**					
Cwa (Warm-temperate/winter dry/hot summer)	6 (8%)	12 (15%)	53 (66%)	9 (11%)	85
Cfa (Warm-temperate/fully humid/hot summer)	0 (0%)	11 (37%)	14 (47%)	5 (17%)	30
Csa (Warm-temperate/summer dry/hot summer)	0 (0%)	0 (0%)	2 (100%)	0 (0%)	2
**Snow**					
Dwa (Snow/winter dry/hot summer)	0 (0%)	2 (67%)	1 (33%)	0 (0%)	3
Dsb (Snow/summer dry/warm summer)	0 (0%)	0 (0%)	1 (100%)	0 (0%)	1

Row percentages are reported.

### Seasonal Patterns in Case Incidence

**Figure 3:**
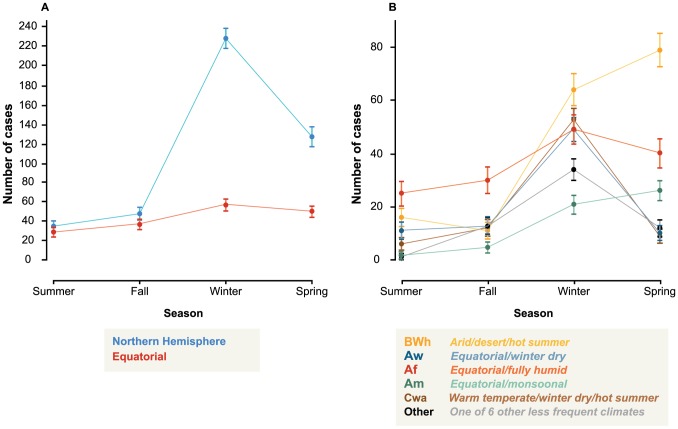
Seasonal highly pathogenic avian influenza H5N1 case occurrence by hemisphere and climate. Error bars represent ± SE estimated via bootstrapping.

Figure 3a displays case incidence by season and hemisphere. Across all countries, 76% of all cases occurred in winter and spring. In the winter and spring periods combined (n = 462), 3.1 times as many cases occurred as in the summer and fall months combined (n = 149). Among non-equatorial countries, 81% of cases occurred in winter and spring periods combined (n = 355), representing a 4.3-fold increase over summer and fall months combined (n = 83).

As expected, a classical Poisson model was overdispersed (residual deviance  = 1221.98, df = 60, p<0.001; also see Figure S1) [20] and yielded artificially low p-values. The negative binomial model appeared to resolve the poor fit due to overdispersion (residual deviance  = 65.40, df = 60; p = 0.29). Table 3 displays regression results for Models (1)–(3). Based on Model (1), case occurrence is higher in winter vs. summer (IRR = 4.45, p = 0.03) and marginally higher in spring vs. summer (IRR = 2.77, p = 0.13), but not in fall vs. summer (IRR = 1.33, p = 0.68).

**Table 3:** Negative binomial regression seasonal occurrence models.

	Model 1		Model 2		Model 3	
	IRR [95% CI]	p value	IRR [95% CI]	p value	IRR [95% CI]	p value
**Season**						
Summer	*Ref*	*Ref*	*Ref*	*Ref*	*Ref*	*Ref*
Fall	1.33 [0.33, 5.29]	0.68	1.34 [0.35, 5.23]	0.66	1.37 [0.30, 6.27]	0.67
Winter	4.45 [1.13, 17.56]	0.03	5.29 [1.36, 20.61]	0.01	6.51 [1.46, 29.21]	0.01
Spring	2.77 [0.70, 10.93]	0.13	3.11 [0.80, 12.08]	0.09	3.63 [0.81, 16.34]	0.08
**Hemisphere**						
Northern	—	—	*Ref*	*Ref*	*Ref*	*Ref*
Equatorial	—	—	2.32* [0.78, 9.05]	0.16	3.59* [0.47, 89.26]	0.28
**Hemisphere** * **season**						
Equatorial-Fall	—	—	—	—	0.93 [0.02, 38.28]	0.97
Equatorial-Winter	—	—	—	—	0.30 [0.01, 12.28]	0.48
Equatorial-Spring	—	—	—	—	0.48 [0.01, 19.39]	0.66
*Model AIC*	367.90		367.74		373.08	
*LR test vs. Model 1*	—		p = 0.14		p = 0.59	

IRR  =  incidence rate ratio, calculated by exponentiating regression coefficient.

*: The IRR for the Equatorial group exceeds 1 despite the lower total frequency of cases among these countries (Fig 2a). This arises because, due to the large number of cases in equatorial Indonesia, the estimated rate of case occurrence is higher among Equatorial countries. However, because there were only three Equatorial countries, the total case frequency remains lower than in the more numerous Northern-hemisphere countries.

**Figure 4:**
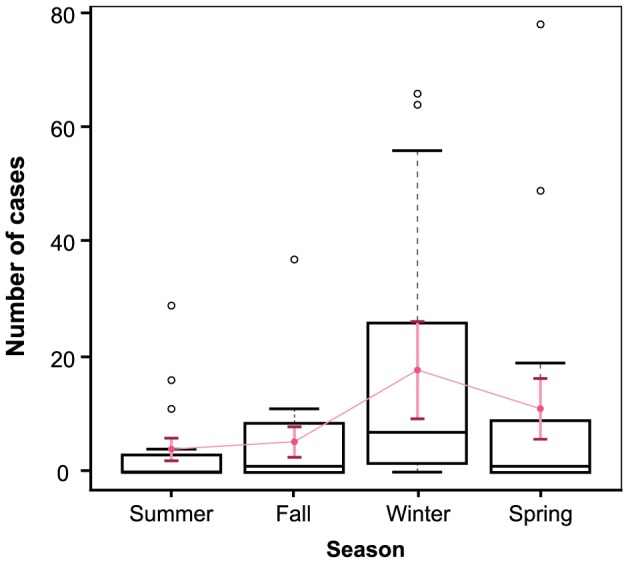
Observed versus fitted highly pathogenic avian influenza H5N1 case occurrence by season. Boxplots represent the observed distribution of HPAI H5N1 case occurrence conditional on season. Overlaid in pink, point estimates (± SE) represent predictions fitted by negative binomial regression (Table 3, Model 1).

Model (2), incorporating a main effect of hemisphere, and Model (3), incorporating an interaction of season with hemisphere, were not significantly better-fitting than model (1) (likelihood-ratio test: chi-square  = 2.16, df = 1, p = 0.14 and chi-square  = 2.82, df = 4, p = 0.59, respectively). Model (1) had comparable AIC (367.90) to Model (2) (367.74) and lower AIC than Model (3) (373.08). Thus, the season-only model adequately described seasonal variation; incorporating a main effect or interaction with hemisphere did not significantly improve fit. Model fit is summarized in Figure 4.

Figure 3b displays case incidence by season and climate. By visual inspection, the winter peak in case occurrence relative to summer and fall is robust across all six climate types. Case frequency in the spring appears more variable by climate group: three climate types (Arid/desert/hot summer, Equatorial/fully humid, and Equatorial/monsoonal) show some increase in frequency in spring relative to summer and fall, while the remaining three (Warm temperate/winter dry/hot summer, Equatorial/winter dry, and Other) do not show a spring peak in case occurrence.

Interactive Map S1 visually depicts the relationship between cases and temperature and humidity over the course of several years in Egypt and Indonesia, the two countries with the highest frequency of cases.

## Discussion

We compiled our dataset of 611 cases through a systematic search of all published human cases of HPAI H5N1, then investigated seasonal incidence patterns in human infections with HPAI H5N1 viruses and explored hemisphere as a potential moderator of seasonal fluctuations in case incidence. We find that the occurrence of human cases of HPAI H5N1 worldwide is more than three times higher during winter and spring periods combined than in fall and summer months combined. The winter peak in case occurrence persists across 15 countries occupying six diverse climate groups.

These robust seasonal differences in the frequency of human HPAI H5N1 virus infections mirror previous research on virus isolate patterns in avian vectors. Surveillance data indicate that the yield of HPAI H5N1 viruses among aquatic and terrestrial poultry in mainland China is highest during winter [7], [8], and space-time cluster analyses show that worldwide HPAI H5N1 outbreaks in wild waterfowl peak in winter and early spring (October–March) [21]. These HPAI H5N1 outbreaks were associated with migration patterns of wild birds and may contribute to the strong seasonal occurrence pattern in human HPAI H5N1 cases. Our results are also consistent with prior research showing improved influenza virus transmission in cold and dry conditions, such as those characterizing winter weather in many climates [22], [23]. Finally, given known associations between human case occurrence and local poultry outbreaks, our findings may reflect seasonal variations in human-poultry contact, for example in live markets and agriculture [24].

Our research has limitations. Standards of surveillance and reporting of HPAI H5N1 cases vary by country and locality, potentially introducing sampling bias [25], [26]. Also, given the higher occurrence of infection with H5N1 in poultry in the winter, surveillance may be more rigorous in these high-risk months. Case reports in the literature were of inconsistent quality and almost never followed standardized reporting guidelines such as the WHO Clinical Case Summary Form [27]. Since we were interested in the illness onset of human cases, and only sporadic clusters of limited, non-sustained human-to-human transmission have occurred to date, we did not distinguish between transmission modality (zoonotic or human-to-human).

Additionally, we find that hemisphere does not significantly moderate the relationship between season and case incidence. However, as most human cases of HPAI H5N1 to date have occurred in the Northern Hemisphere and no cases have been reported in the Southern Hemisphere, fully assessing the role of hemisphere was not possible, and power to test the interaction was inherently limited due to the relatively small number of cases and affected countries. Another possible limitation is the regression assumption that cases occur independently; however, for a rare disease such as HPAI H5N1 occurring in geographically- and temporally-separated regions, any violation of this assumption is likely inconsequential.

We defined climate types using average regional climate patterns, but were not able to model day-by-day local weather fluctuations that may influence HPAI H5N1 viral transmission. A promising direction for future research, therefore, is to develop finer-grained incidence models of climate incorporating local weather at the time of infection. Modeling factors known to alter influenza virus transmission, such as humidity and temperature, would likely improve predictive power and elucidate the role of climate. Future research could also investigate other possible mechanisms for the observed seasonal patterns, such as variations in poultry industry and trade activities.

To our knowledge, our work represents the most comprehensive analysis of global seasonal patterns in all known human HPAI H5N1 cases to date. We found robust peaks in case occurrence during winter and spring months. Prioritizing prevention and control activities among poultry and targeting public health messaging to reduce poultry exposures during winter and spring months may help to reduce zoonotic transmission of HPAI H5N1 virus in resource-limited settings. Contingent on viral sampling in poultry markets and continuous veterinary surveillance, the public could be advised on the relatively lower risk of visiting live poultry markets during periods of the year with lower case occurrence. Additionally, in countries with existing poultry vaccination programs, such as Egypt, a well-matched poultry vaccine could be administered strategically before peak seasonality.

## Supporting Information

Figure S1 **Distribution of case frequency by season.** Unit of analysis is the number of cases occurring in a given country in a given season. Thus, each season-conditioned histogram above contains n = 16 data points, one for each country. The unconditional histogram in the bottom panel contains n = 64 data points, representing 4 seasons and 16 countries.
(EPS)

Abstraction Form S1 (DOCX)

Map S1 **Interactive visualization of spatiotemporal case occurrence and local weather.** Data presented in circles are temperatures (degrees F) and percent humidity. Blue dots represent case occurrences. Weather data provided by Wunderground.com.
(ZIP)
