# Supplementary material for: Seasonal Patterns in Human A (H5N1) Virus Infection: Analysis of Global Cases
Source: PLoS One. 2014 Sep 12;9(9):e106171. doi: 10.1371/journal.pone.0106171 (PMC4162536; doi:10.1371/journal.pone.0106171)
Supplement: Abstraction Form S1 — (DOCX) [file pone.0106171.s002.docx]

# SUPPORTING INFORMATION

***Abstraction form S1***

# ABSTRACTION INFORMATION

**Reviewer Date:** _____________________

- MM
- RP
- NK

# CITATION INFORMATION

**Search Engine:** _________________________ **Reference #:** ___________________________________

**Title:** __________________________________________________________________________________________________________

**Journal:** ______________________________________________________________________________________________________

**Year:** ___________

**Authors:** _____________________________________________________________________________________________________

**Institution(s):** ______________________________________________________________________________________________

**Relevant References:** _____________________________________________________________________________________

__________________________________________________________________________________________

__________________________________________________________________________________________

# LANGUAGE

- English
- Other (describe language and translator): ____________________________________________________

| **ARTICLE INCLUSION/EXCLUSION RATIONALE** | |
| --- | --- |
| **Does this article meet inclusion criteria?** | |
| **YES. Requires all categories to meet criteria:**   - Human subject - Individual case data available (including meta-analysis articles) - Laboratory-confirmed influenza A (H5N1) virus OR suspected H5N1 case amongst known case cluster. - H5N1 culture *or* - H5N1 PCR study *or* - Antibody titer 1:80 | **NO**. Does not meet criteria for these reasons:   - Non-human: molecular or animal - Individual case details not provided - Insufficient epidemiological data |

**COMPLETE THE FOLLOWING IF THE ARTICLE MET INCLUSION CRITERIA:**

| **STUDY DESIGN INFORMATION** |
| --- |
| **Describe the study design:** |

| **PRE-ADMISSION PREDICTOR VARIABLES DESCRIBED** | |
| --- | --- |
| **Demographic variables:**   - Country where A (H5N1) virus infection identified - Per capita government expenditure on health (PCGEH) - Season - Age - Sex - Body Mass Index (BMI) - Co-morbidities | **Infection-related variables:**   - Case is part of a cluster of known cases - Contact with poultry prior to symptom onset - Delay from symptom onset to hospitalization (days) |

| **POST-ADMISSION PREDICTOR VARIABLES DESCRIBED** | |
| --- | --- |
| **Hospitalization variables:**   - Laboratory data - Presence of pneumonia - Acute respiratory distress syndrome (ARDS) - Mechanical ventilation |  |

| **OUTCOME VARIABLES DESCRIBED** |
| --- |
| - Mortality |

| **ADDITIONAL COMMENTS** |
| --- |
|  |

***Figure S1:* Distribution of case frequency by season**

*Unit of analysis is the number of cases occurring in a given country in a given season. Thus, each season-conditioned histogram above contains n=16 data points, one for each country. The unconditional histogram in the bottom panel contains n=64 data points, representing 4 seasons and 16 countries.*

***Map S1:* Interactive visualization of spatiotemporal case occurrence and local weather**

*Data presented in circles are temperatures (degrees F) and percent humidity. Blue dots represent case occurrences. Weather data provided by Wunderground.com.*
