# Supplementary material for: Seasonal Patterns in Human A (H5N1) Virus Infection: Analysis of Global Cases
Source: PLoS One. 2014 Sep 12;9(9):e106171. doi: 10.1371/journal.pone.0106171 (PMC4162536; doi:10.1371/journal.pone.0106171)
Supplement: Map S1 — Interactive visualization of spatiotemporal case occurrence and local weather. Data presented in circles are temperatures (degrees F) and percent humidity. Blue dots represent case occurrences. Weather data provided by Wunderground.com. (ZIP) [file pone.0106171.s003.zip › Map S1/Egypt.html]

Untitled
